# miR-4456/CCL3/CCR5 Pathway in the Pathogenesis of Tight Junction Impairment in Chronic Obstructive Pulmonary Disease

**DOI:** 10.3389/fphar.2021.551839

**Published:** 2021-04-19

**Authors:** Weiwei Yu, Ting Ye, Jie Ding, Yi Huang, Yang Peng, Qin Xia, Zhang Cuntai

**Affiliations:** ^1^ Department of Geriatric Medicine, Tongji Hospital, Tongji Medical College, Huazhong University of Science and Technology, Wuhan, China; ^2^ Department of Clinical Nutrition, Tongji Hospital, Tongji Medical College, Huazhong University of Science and Technology, Wuhan, China; ^3^ Urology Department of Xin Hua Hospital, Xin Hua Hospital Affliated to Shanghai Jiao Tong University, Shanghai, China

**Keywords:** tight junctions, miR-4456, C-C chemokine receptor type 5, chemokine (C-C motif) ligand 3, chronic obstructive pulmonary disease

## Abstract

**Background:** Cigarette smoke exposure (CSE) is a major cause of chronic obstructive pulmonary disease (COPD). The smoke disrupts cell-cell adhesion by inducing epithelial barrier damage to the tight junction (TJ) proteins. Even though the inflammatory mechanism of chemokine (C-C motif) ligand 3 (CCL3) in COPD has gained increasing attention in the research community, however, the underlying signaling pathway, remains unknown.

**Objectives:** To identify the relationship of CCL3 in the pathogenesis of tight junction impairment in COPD and the pathway through which CSE causes damage to TJ in COPD via CCL3, both *in vivo* and *in vitro*.

**Methods:** We screened the inflammatory factors in the peripheral blood mononuclear cells (PBMCs) from healthy controls and patients at each GOLD 1-4 stage of chronic obstructive pulmonary disease. RT-PCR, western blot, and ELISA were used to detect the levels of CCL3, ZO-1, and occludin after Cigarette smoke exposure. Immunofluorescence was applied to examine the impairment of the TJs in 16-HBE and A549 cells. The reverse assay was used to detect the effect of a CCR5 antagonist (DAPTA) in COPD. In the CSE-induced COPD mouse model, H&E staining and lung function tests were used to evaluate the pathological and physical states in each group. Immunofluorescence was used to assess the impairment of TJs in each group. ELISA and RT-PCR were used to examine the mRNA or protein expression of CCL3 or miR-4456 in each group.

**Results:** The *in vivo* and *in vitro* results showed that CCL3 expression was increased in COPD compared with healthy controls. CCL3 caused significant injury to TJs through its C-C chemokine receptor type 5 (CCR5), while miR-4456 could suppress the effect of CCL3 on TJs by binding to the 3′-UTR of CCL3.

**Conclusion:** miR-4456/CCL3/CCR5 pathway may be a potential target pathway for the treatment of COPD.

## Introduction

COPD is characterized by progressive, poorly reversible airflow obstruction associated with an abnormal inflammatory response to environmental exposure. Tobacco smoking causes a self-maintaining inflammatory process that is considered as a critical factor in the pathophysiology of COPD (Barnes et al., 2015). Inhaled tobacco smoke first reaches the airway epithelium, which represents a highly regulated barrier (Hammad and Lambrecht 2015). Epithelial physical barriers are maintained by various intercellular junctions. The tight junctions (TJs), which are comprised of the interacting proteins such as occludin, ZO-1 and claudins (Zihni et al., 2016; Buckley and Turner 2018). Occludin is found at TJs and involved in the formation, maintenance, and function of TJs (Furuse et al., 1993; Zihni et al., 2016), and claudins are tightly bound to the cell membrane and are important components of TJs (Ruffer and Gerke 2004; Zihni et al., 2016). ZO-1 is also critiacl component of the TJs, where it plays roles in signal transduction at the cell-cell junction (Itoh et al., 1997; Zihni et al., 2016). Numerous studies have shown that the TJs of airway epithelium are involved in the pathogenesis of COPD (Roscioli et al., 2017). For instance, Smoking may considerably disturb epithelial junctions by inducing structural changes in the airways of patients with COPD, such as mucous hyperplasia (Gohy et al., 2016). Therapeutic strategies that attenuate TJ damage during inflammation and/or support TJs restoration have been shown to improve the clinical outcomes in COPD patients (Wittekindt 2017). Smoking causes the delocalization of ZO-1 and occludin from the cell-cell boundaries and a subsequent loss of epithelial integrity (Azghani 1996; Mankertz et al., 2000; Olivera et al., 2010; Yadav et al., 2013). Nevertheless, the underlying mechanisms of how TJs were damaged thus causing barrier dysfunction are still not fully understood.

CCL3, also known as macrophage inflammatory protein-1α (MIP-1α), is a monocyte and macrophage chemoattractant (Larsson 2008). There existed evidence that CCL3 levels increase in bronchial epithelial cells of COPD patients (Villanueva and Llovet 2011). CCL3 is also potentially an important genetic regulator of T-lymphocytes, macrophages, and chemoattractants for mononuclear cells (Larsson 2008). CCR5, the receptor of CCL3, was reported to increase the numbers of macrophages and T-cells in the lungs of patients with COPD, And the inhibition of CCR5 was considered as a viable treatment to reduce the inflammatory response COPD (Wang and He 2012; Costa et al., 2016; Ravi, Plumb et al., 2017). The expression level of CCR5 in inflammatory cells from induced sputum was potentially associated with COPD severity (Wang and He 2012). CCL3 also played an important role in promoting the TJs injury in lung epithelial by binding to CCR5 (Polianova et al., 2005; Camargo et al., 2009; Li et al., 2016; Ahmad et al., 2019). The upregulation of CCL3 might facilitate the recruitment of macrophages into the airways since CCR1 and CCR5, the receptors for CCL3, participate together in macrophage recruitment (Ravi et al., 2014). Using a CCR5 antagonist could attenuate aberrant immune responses (Ahmad et al., 2019), thus protecting against ischemia-reperfusion injury (Li et al., 2016) while overexpressing CCR5 will lead to enhance IL-2 production by T cells (Camargo et al., 2009). The exact regulatory mechanism of CCL3 and CCR5 in COPD pathogenesis remained unkown.

There have been studies showing the dysregulation and role of microRNAs (miRNAs) in COPD. Van Pottelberge et al. reported that 34 miRNAs were differentially expressed between never-smokers and current smokers without airflow limitation, and eight of them was significantly lower in current-smokers with COPD (Van Pottelberge et al., 2011). Another study showed that miRNA-34c is associated with emphysema severity in COPD (Francis et al., 2014). Moreover, miRNAs have been shown to regulate transforming growth factor (TGF)-β, Wnt, and focal adhesion pathways, thus suggesting that they might also involved in the pathogenesis of COPD (Ezzie et al., 2012). In human bronchial airway epithelium, the expression of miRNAs was largely affected by smoking, among them, most miRNAs were down-regulated in current smokers (Schembri et al., 2009). In the epithelial cells of intestine and urethra, miRNAs exhibited vital roles in the barrier function of intestinal epithelial cells and urethra epithelial cells (Ikemura et al., 2014; Chung et al., 2018). Specifically, many genes that associated with epithelial TJ barrier permeability such as occludin, tumor necrosis factor (TNF)-α, and HIF1α (Ikemura et al., 2014; Kar et al., 2017) were regulated. miR-21 might regulate intestinal epithelial TJ permeability through the PTEN/PI3K/Akt signaling pathway (Zhang et al., 2015).

Therefore, we hypothesized that specific microRNAs played key roles in the epithelial TJ of COPD by modulating the CCL3/CCR5 axis. The aim of the present study was to elucidate the role and miRNAs regulation mechanism of CCL3 in the pathogenesis of tight junction impairment correlated COPD. The present study also investigated the relationship between miRNA and epithelial TJ and found the target miR-4456, which could regulate the CCL3/CCR5-induce impairment of TJ in COPD.

## Materials and Methods

### Study Subjects

COPD patients were categorized according to the GOLD (Vogelmeier et al., 2017). Peripheral blood mononuclear cells (PBMCs) were obtained from subjects with normal lung function [non-smokers (NS); eight subjects] and 40 patients with mild to severe COPD (stage 1, nine subjects; stage 2, nine subjects; stage 3, 10 subjects; and stage 4, 12 subjects). The participants had no history of allergy (negative IgE tests) or asthma, did not use inhaled or oral corticosteroids, and had no exacerbations for >3 months prior to study inclusion.

The ethics committee of Tongji Hospital, Tongji Medical College of Huazhong University of Science and Technology approved this study, and informed written consent was obtained from all subjects (Ethical consent for clinical trials. No:WDWHTZKJTJ-0123566). The clinical features of the patients and healthy controls are shown in Table 1.

**TABLE 1:** Clinical characteristics of the subjects involved in the studies.

Characteritics	NS	GOLD1	GOLD2	GOLD3	GOLD4
Number of subjects	8	9	9	10	12
Age (years)	63.2 ± 11.2	61.1 ± 13.2	64.5 ± 15.8	68.3 ± 12.2	62.3 ± 10.5
Sex, male (female)	7 (1)	8 (1)	7 (2)	8 (2)	10 (2)
Pack-years	0	15.3 ± 4.5*	34.8 ± 9.5**	32.5 ± 4.7**	32.5 ± 4.7**
FEV1 (% predicted)	90.3 ± 6.2	63.7 ± 5.3	54.2 ± 5.3*	43.4 ± 3.5**	41.1 ± 2.5**
FEV1/FVC (%)	83.4 ± 5.9	62.3 ± 7.3	50.9 ± 4.9*	40.1 ± 2.1**	38.4 ± 3.1**

** Notes: Values are expressed as mean ± SD. *p < 0.05, *p* < 0.01, * compared with NS. Abbreviations: NS, no smoke; FEV1 (% predicted), forced expiratory volume in 1 s as percentage of percentage of predicated value; FVC, forced vital capacity.

### PBMCs Isolation and RNA Extraction

PBMCs were isolated from venous blood by density gradient centrifugation using Ficoll-Paque PLUS (GE Healthcare, Uppsala, Sweden) and suspended in QIAzol lysis reagent (Qiagen, Dusseldorf, Germany). Total RNA was extracted using the miRNeasy Mini Kit (Qiagen) according to the manufacturer’s procedure. RNA integrity was determined by formaldehyde denaturing gel electrophoresis.

### Human Cytokine Array

Protocol followed manual instructions from R&D Systems Europe, Ltd., Human Cytokine Array (#ARY005B). Briefly, cell lysates of PBMCs were diluted and incubated overnight with either array. The array was washed to remove unbound proteins followed by incubation with a cocktail of biotinylated detection antibodies and with streptavidin-HRP antibodies. Captured signal corresponded to the amount of bound phosphorylated protein. The R software (version 3.2.0) was used for further cluster analysis.

### Cell Culture and CSE Treatment

The 16-HBE cell line was purchased from the American Type Culture Collection (ATCC, Manassas, VA, United States). A549 cells were kindly provided by D.C. Shuyuan Yeh (University of Rochester, Rochester, NY, United States). The human bronchial epithelial cells BEpic (CS1028Hu01) and human alveolar epithelial cells (CS1093Hu01) were purchased from Wuhan Cloud-Clone Co., Ltd. (Wuhan, China). The cells were cultured in F-12K medium added with 10% fetal bovine serum (GIBCO, Invitrogen Inc., Carlsbad, CA, United States) (Marcos-Vadillo and Garcia-Sanchez 2016a; Marcos-Vadillo and Garcia-Sanchez 2016b). Before experimentation, cell viability was evaluated by Trypan blue staining (mean viability 95 ± 0.6% for brushed cells and 93 ± 1.6% for Lonza cells). CSE was freshly prepared on the day of the experiment. In brief, the smoke generated from two burning cigarettes (Red Roses Label; tar, 13 mg; nicotine, 1.3 mg) without filters was sucked under a constant flow rate (50 ml/10 s) into a syringe and then bubbled into a tube containing 10 ml of serum-free DMEM medium. The CSE solution was sterilized using a 0.22 µm filter (Millipore, Bedford, MA, United States), and the pH was adjusted to 7.4. This CSE solution was considered as 100% CSE. The cells were treated with 0 and 1% CSE concentrations for 24 h, respectively. The cells treated with 0 and 1% CSE were the control and CSE treatment groups, respectively. Cells pretreated with DAPTA (0.1 mM) served as CSE + DAPTA treatment group. After treatment of CSE, the cells were washed with serum free RPMI and were treated with DAPTA at the indicated concentrations for 6 h at 37 C, 5% CO2. Cultures were washed two times to remove unabsorbed DAPTA. The dosage of DAPTA was based on the literature (Polianova, Ruscetti et al., 2005). There were three wells in each group.

### RT-PCR Analysis

Total RNA (1 µg) was subjected to reverse transcription using the Superscript III transcriptase (Invitrogen, Grand Island, NY, United States). Quantitative real-time PCR (qRT-PCR) was conducted using a Bio-Rad CFX96 system with SYBR green to determine the mRNA expression levels of a gene of interest. Expression levels were normalized to the expression of β-actin. miRNAs were isolated using the PureLink® miRNA kit. Briefly, 50 ng of RNA was processed for poly-A addition by adding one unit of polymerase with 1 mM ATP in 1× RT buffer at 37°C for 10 min in 10 μl, and heat inactivating at 95°C for 2 min. Next, 50 mM of anchor primer was added to a total of 12.5 μl and incubated at 65°C for 5 min. cDNA synthesis was performed by adding 2 μl of 5× RT buffer, 2 μl of 10 mM dNTP, and 1 μl of reverse transcriptase was added to a total of 20 μl, and the sample was incubated at 42°C for 1 h. Quantitative real-time PCR (qRT-PCR) was conducted using a Bio-Rad CFX96 system with SYBR green to determine the mRNA expression level of a gene of interest. The expression levels were normalized to the expression of 5S RNA and/or U6.

### Western Blotting

The cells were lysed in RIPA buffer. Proteins (30 µg) were separated by 8–10% SDS/PAGE and transferred onto PVDF membranes (Millipore, Billerica, MA, United States). After blocking, the membranes were incubated with the appropriate dilutions of specific primary antibodies against ZO-1 (1:200, cat#: pa5-28858, Thermo Fisher Scientific, Rochester, NY, United States) occludin (1:200, cat#: ab216327, Abcam, Cambridge, MA, United States), Claudin (1:200, cat#:ab180158, Abcam, Cambridge, MA, United States), CCL3 (1:1000, cat#: ab229900, Abcam, Cambridge, MA, United States), and CCR5 (1:200, cat#: ab110103, Abcam, Cambridge, MA, United States). The blots were next incubated with HRP-conjugated secondary antibodies (1; 1000, cat#: a12004-1, Gepigentek, Farmingdale, NY, United States) and visualized using the ECL system.

### ELISA

Analysis of the CCL3 levels was carried out based on the enzyme-linked immunosorbent assay with the Human CCL3 Quantikine ELISA Kit (cat#: SMA00, R&D, Minneapolis United States) according to the manufacturer’s instructions.

### Transepithelial Electrical Resistance (TER) Measurement

The 16HBE or A549 cells were seeded in the upper chamber of a Transwell tissue culture plate (12 mm diameter, 0.4 μm pore size, Costar, Corning Inc., Corning, NY, United States) and allowed to reach confluence. The basolateral and apical sides of the filters were exposed to CCL3 (10 mg/ml) when indicated. The TER of the cells grown on filters was measured after 7 days, with an epithelial volt-ohm meter (Endohm; World Precision Instruments, Sarasota, FL, United States). To explore the rapid effect of CCL3 on the TER, the volt-ohm meter was coupled to an A/N converter (World Precision Instruments, Sarasota, FL, United States), and the TER measurement was monitored using Powerlab software (Chart for Windows, v4.0, AD Instruments, Sydney, Australia) with an acquisition frequency of 2 Hz. The background electrical resistance attributed to fluid and a blank Transwell filter were subtracted from the measured TER. The TER measurements were normalized by the area of the monolayer and given as cm^2^. Untreated 16HBE cells have been reported to have a TER around 600 Ω. cm^2^ (Yuan et al., 2020), while A549 cells have been reported to have a TER around 175 Ω. cm^2^ (Albano et al., 2020).

### Animal Studies

C57BL/6 mice, 6–8 weeks old, weighing 18–25 g, were obtained from Tongji Medical Laboratory Animal Center (Wuhan, China). All animals were housed in an environment with a temperature of 22 ± 1°C, relative humidity of 50 ± 1%, and a light/dark cycle of 12/12 h. All animal studies (including the mice euthanasia procedure) were carried out in compliance with the regulations and guidelines of Huazhong University institutional animal care and conducted according to the AAALAC and the IACUC guidelines (Animal Ethical consent No:SYXK2017-0023).

The C57BL/6 mice were randomized into following groups (*n* = 8 for each group): 1) control group: exposed to normal air, then subcutaneously injected with PBS, 10 ml kg^−1^, 2) CCL3 group: exposed to normal air, then subcutaneously injected with CCL3 (cat#: csb-ap001221monthsnth, 200 ng kg^−1^, 0.01 mg ml^−1^ dissolved in normal saline, CUSABIO, Wuhan, China) for 6 weeks, 3) CSE group: inhalation of CS for up to 12 weeks as previously described (Li et al., 2018a; Li et al., 2018b), and each exposure lasted for 75 min, 4) CSE + DAPTA group: mice were chronically exposed to CS for 12 weeks, then subcutaneously injected with DAPTA (cat#: ab120810, Abcam, Cambridge, MA, United States; l0 μg kg^−1^, 0.01 mg ml^−1^ dissolved in normal saline) 15 min before the first CS-exposure on each day, starting at 6 weeks (Li et al., 2016). 5) CSE + miR-4456 group and CSE + miR-NC group: mice were chronically exposed to CS for 12 weeks, then 120 nM/kg miR-4456 agomir or miR-NC was injected via the tail vein weekly over the next four weeks. The mice were sacrificed on day 56 following CSE/CCL3 administration. The sequence of agomir-miR-4456 was: cuc​ugg​aau​cau​cau​guc​aca​ga (double-stranded); the sequence of the miR-NC was: uuc​ucc​gaa​cgu​guc​acg​u (double-stranded). Lung tissues were harvested, quick-frozen in liquid nitrogen and stored at −80 C immediately for further analysis.

### Lung Function Measurement

The modeling efficiency was evaluated by lung function, including airway resistance, elasticity, static compliance. Lung function was evaluated as previously described (Zhuang, Huang et al., 2016; (Irvin and Bates 2003). The rats undergoing non-invasive pulmonary function were monitored by whole-body barometric plethysmography (WBP; EMKA Technologies, Paris, France). Rats were placed in a plethysmograph chamber, and a 10 min accommodation was allowed before analysis. Respiratory parameters were recorded while the rats were unrestrained, and the respiratory frequency (F) and tidal volume (TV) were analyzed by emka Technologies iox2 software.

### H&E Staining

The whole lungs were fixed in 4% neutral buffered paraformaldehyde and embedded in paraffin. Tissues were cut into 5 µm sections and analyzed using H&E staining.

### Bronchoalveolar Lavage

Following lung mechanical measurements, the animals were detached from the ventilator and sacrificed by exsanguination (inferior vena cava and descending aorta dissection). The left main bronchus was temporarily ligated, and the right lung was lavaged with three aliquots of 2.5 ml of normal saline. Bronchoalveolar lavage fluid (BALF) was withdrawn and immediately centrifuged at 300 ×g for 10 min at 4°C. The supernatant was collected and stored at −80°C, while the cell pellet was resuspended in 1 ml of normal saline. Total protein concentration in BALF was measured using a colorimetric protein assay according to the manufacturer’s instructions (Bio-Rad Laboratories Inc., Hercules, CA, United States). Bovine serum albumin was used to create standard curves.

### Immunofluorescence Microscopy

The cells were first fixed in 100% methanol for 5 min at room temperature and then incubated with 1% BSA in Ca^2+^− and Mg^2+^−free PBS (PBS(−)) for 1 h at room temperature. After incubation for 2 h with ZO-1 antibody (1:100, cat#:pa5-28858, Thermo Fisher, Waltham, MA, USA) or occludin antibody (1:100, ab216327, Abcam, Cambridge, MA, United States) for 1 h at 37 C, washed with PBS(−), the cells were incubated for 1 h with Alexa Fluor 488-conjugated secondary antibodies HRP (1:2000, cat#:ab205718, Abcam, Cambridge, MA, United States) or H&L (1:2000, cat#:ab150077, Abcam, Cambridge, MA, United States). The results were examined with a fluorescence microscope (Olympus BX51; Olympus, Tokyo, Japan).

Mouse lungs in the thoracic cages were infused through the trachea with 60% optimal cutting temperature compound (Tissue-Tek; Miles Laboratories, Elkhart, IN, United States) in PBS, removed, and frozen in liquid nitrogen. The tissues were cut into 10 μm-thick frozen sections using a cryostat. For immunofluorescence staining, the lungs were fixed with ice-cold 95% ethanol, followed by 100% acetone at room temperature for 1 min, and then washed three times in PBS. Cultured cells were fixed with 3% formaldehyde for 15 min, followed by 0.1% Triton X-100 for 3 min at room temperature, and washed three times in PBS. After soaking in PBS containing 3% BSA, the sections were incubated with primary antibodies in a moist chamber for 1 h. They were washed three times with PBS and incubated for 30 min with secondary antibodies and 4,6-diamino-2-phenylindole for nuclear staining. The samples were washed with PBS and observed under a fluorescence microscope (Olympus BX51; Olympus, Tokyo, Japan).

### Cytokine Levels in Lung Tissue Using ELISA

Frozen lung tissue sections were homogenized with a buffer containing 50 mM HEPES (pH 7.5), 150 nM NaCl, 10% glycerol, 1% Triton X-100, 1 mM EDTA, 1.5 mM MgCl_2_, and a cocktail of protease and phosphatase inhibitors at a 1:1000 concentration. The samples were centrifuged at 10,000 ×g for 10 min. The supernatant was collected, and total protein concentration was estimated using a colorimetric protein assay according to the manufacturer’s instructions. Protein levels of interleukin IL-6, IL-18 and TNF- α were determined in lung tissue homogenates using ELISA, according to the manufacturer’s protocol (DuoSet ELISA; R&D Systems, Inc., Minneapolis, MN, United States) and normalized to the total protein content of lung homogenates. Oxidative stress was evaluated based on the levels of SOD, CAT, and GSH-Px using ELISA, according to the manufacturer’s protocol (DuoSet ELISA; R&D Systems, Inc., Minneapolis, MN, United States) and normalized to the total protein content of lung homogenates.

### Statistical Analysis

Data are expressed as means ± standard deviations from at least three sets of independent experiments performed in triplicate (*n* = 3/experiment). The data were checked for normal distribution using the Kolmogorov-Smirnov test and were log-transformed to normalize their distribution when needed. Statistical analyses involved Student’s t-test, one-way ANOVA, and the log-rank (Mantel-Cox) test with SPSS 22 (IBM Corp, Armonk, NY, United States) or GraphPad Prism 6 (GraphPad Software, Inc., La Jolla, CA, United States). *p* < 0.05 was considered statistically significant.

## Results

### Higher Expression of CCL3 in the PBMCs of COPD

**FIGURE 1:**
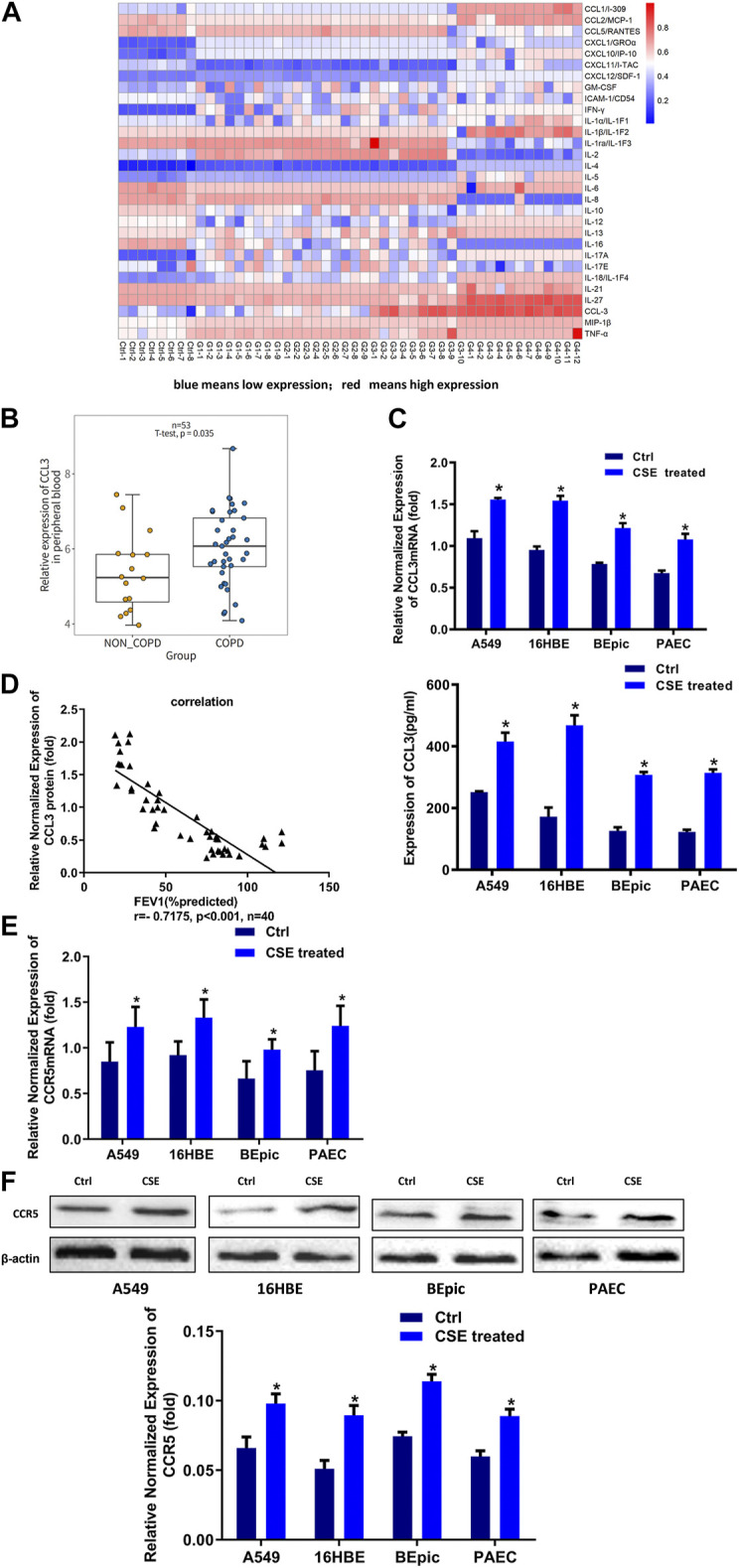
Higher expression of CCL3 in the supernatants of PBMCs cells **(A)** Human Cytokine Array for the parallel determination of relative levels of cytokines and chemokines in the supernatants of PBMCs cells. Downregulated proteins are shown in blue, and upregulated proteins are shown in red, as the mean of all specimens included (*n* = 48). non-smokers: Ctrl-1 to Ctrl-8, GOLD 1:G1-1 to G1-9, GOLD2: G2-1 to G2-9, GOLD3:G3-1 to G3-10, GOLD4:G4-1 to G4-12 **(B)** Expression of CCL3 in no-COPD and COPD patients in the human GEO database (*n* = 53) **(C)** Real-time PCR assays were performed in 16HBE, A549, BEpic, and PAEC cells to detect CCL3 mRNA expression before and after CSE treatment. *n* = 3, *p < 0.05, vs. the control group (left panel). The expression levels were normalized to the expression of GAPDH. ELISA assays were performed in 16HBE, A549, BEpic, and PAEC cells to detect CCL3 protein expression before and after CSE treatment. *n* = 3, *p < 0.05, vs. the control group (right panel) **(D)** Correlation between FEV1 (%predicted) and CCL3 expression in patients with COPD. *r* = 0.7175, *p* < 0.001, *n* = 40 **(E)** Real-time PCR assays were performed in 16HBE, A549, BEpic, and PAEC cells to detect CCR5 mRNA expression before and after CSE treatment. *p < 0.05, vs. the control group **(F)** Western blot assays were performed in 16HBE, A549, BEpic, and PAEC cells to detect CCR5 protein expression before and after CSE treatment. *n* = 3, *p < 0.05, vs. the control group. The expression levels were normalized to the expression of β-actin.

It was well established that PBMCs had a crucial role in COPD.(Bahr et al., 2013). We first screened the inflammatory factors (Inflammatory Factors kits, Roche) in the PBMCs; the level of CCL3 was significantly higher in patients with COPD GOLD 3–4 stage compared with the NS and COPD GOLD 1–2 stage (Figure 1A). Consistantly, the analysis form the human GEO database also showed a significantly higher expression of CCL3 in COPD compared with non-COPD (*p* = 0.0304, *n* = 53; Figure 1B). We then treated alveolar epithelial cells (A549 cells), bronchial epithelial cells (16HBE cells), and primary cells (BEpic and PAEC) with CSE and found that CSE evoked a significantly up-regulation expression of the CCL3 mRNA (*p* < 0.05; Figure 1C, upper) as well as its protein expression (*p* < 0.05) (Figure 1C, lower). We also measured the CCR5 expression, the receptor of CCL3, and found that CSE could also prompt CCR5 mRNA expression (*p* < 0.05; Figure 1E) and CCR5 protein expression (*p* < 0.05) (Figure 1F). Figure 1D revealed that the FEV1 (% predicted) of patients with COPD was negatively correlated with CCL3 protein expression (*p* < 0.001, *n* = 40). Taken together, these results suggested that CSE could promote the expression of CCL3 and CCR5 in COPD.

### CCL3 Promotes Epithelial Tight Junction Injury *via* Binding With CCR5

**FIGURE 2:**
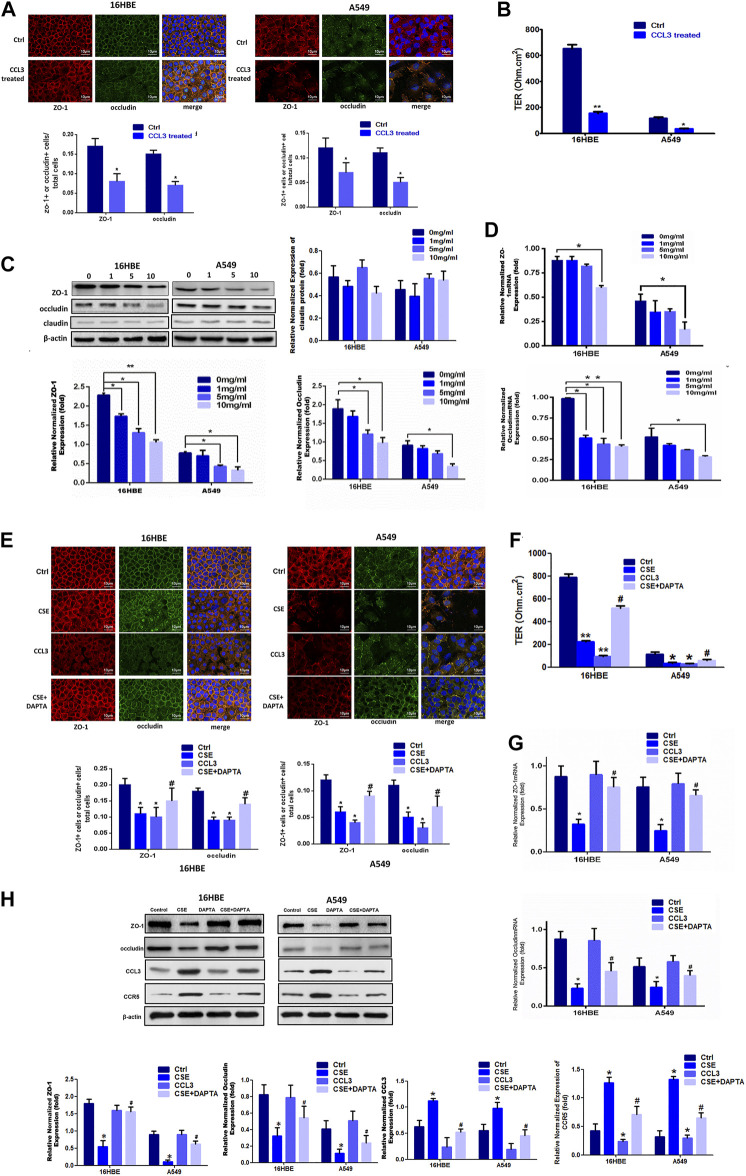
CCL3 promotes epithelial tight junction injury by binding with CCR5 **(A)** Tight junction proteins ZO-1 (red), occludin (green), and merged (blue + orange) were stained in 16HBE cells (left panel) and A549 cells (right panel) by immunofluorescence. Presented as means ± standard error. *n* = 3, *p < 0.05, vs. the control group. Data are representative of three independent experiments. Scale bar = 10 µm **(B)** Transepithelial electrical resistance (TER) after CCL3 treatment (10 mg/ml), as a cell function test 7 days after plating the airway epithelial cells on coated permeable filters. Presented as means ± standard error. *n* = 3, *p < 0.05, vs. the control group **(C)** Western blot analysis to detect protein expression of ZO-1, claudin and occludin in 16HBE cells and A549 cells using different concentrations of CCL3. *n* = 3, *p < 0.05, vs. the control group, **p < 0.01, vs. the control group (CCL3: 0 ng/ml). The expression levels were normalized to the expression of β-actin (left panel). *n* = 3 **(D)** Real-time PCR assays of ZO-1 (upper) and occludin (lower) mRNA expression in 16HBE cells and A549 cells using different concentrations of CCL3. *n* = 3, *p < 0.05, vs. the Ctrl group, **p < 0.01, vs. the control group. Protein expression was normalized to GAPDH **(E)** Tight junction proteins ZO-1 (red), occludin (green) and merged (blue and orange) were stained by immunofluorescence in the four groups: 1) Ctrl (PBS:10 mg ml^−1^), 2) CSE (10 mg ml^−1^), 3) CCL3 (CCL3 10 mg ml^−1^), and 4) CSE (10 mg ml^−1^)+DAPTA (0.1 mg ml^−1^) in 16HBE cells (left panel) and A549 cells (right panel). Presented as means ± standard error. *n* = 3, *p < 0.05, vs. the control group. #*p* < 0.05, vs. the CSE group. Data are representative of three independent experiments. Scale bar = 10 µm **(F)** TER in the four groups same as Fig3E in 16HBE cells (left panel) and A549 cells (right panel). The results are shown as a function test on 7 days after plating the airway epithelial cells on coated permeable filters. Presented as means ± standard error. *n* = 3,*p < 0.05, vs. the control group, **p < 0.01, vs. the control group, #*p* < 0.05, vs. the CSE group **(G)** Real-time PCR assays to detect ZO-1 mRNA (upper panel) and occludin mRNA (lower panel) expression in 16HBE cells and A549 cells in four groups same as Fig3E. *n* = 3,*p < 0.05, vs. the control group, #*p* < 0.05, vs. the CSE group. The expression levels were normalized to the expression of GAPDH (left panel) **(H)** Western blot analysis to detect ZO-1protein, occludin protein, CCL3 protein and CCR5 protein expression in 16HBE cells and A549 cells in four group same as Fig3E. Protein expression levels were normalized to β-actin expression. *n* = 3, *p < 0.05, vs. the control group, #*p* < 0.05, vs. the CCL3 group. Protein expression was normalized to β-actin.

Next, we sought to evaluate the potential roles of CCL3 in COPD. Exogenous application of CCL3 (10 mg/ml) in both 16HBE and A549 cells obviously reduced the epithelial TJs injury when compared with control cells (Figure 2A) and decreased the TER (Figure 2B). Moreover, CCL3 decreased the expression of ZO-1 and occludin, but not claudin, at both the mRNA and protein levels in a concentration-dependent manner (*p* < 0.05) (Figures 2C,D). Previous studies have shown that the expression of CCR5, the critical receptor of CCL3 is higher in patients vs. normal individuals, with the clinical stage (Costa et al., 2016)). We then examined whether CSE induces TJs injury through the CCR5 receptor in 16HBE and A549 cells. We found that CCR5 antagonist (DAPTA, ab120810, 0.1 mM) significantly reduced the CSE-induced TJs injury (Figures 2E,F). Mechanically, DAPTA hampered the CSE reduced expression of ZO-1, occludin, CCL3 and CCR5 in these cells (Figures 2G,H). The increased expression level of CCL3 and CCR5, inversly, were inhibited by DAPTA. Together, our data suggest that CCL3 and CSE promote epithelial tight junction injury via binding with CCR5.

### MiR-4456 Is an Upstream Signal for CCL3-Induced TJ Injury

**FIGURE 3:**
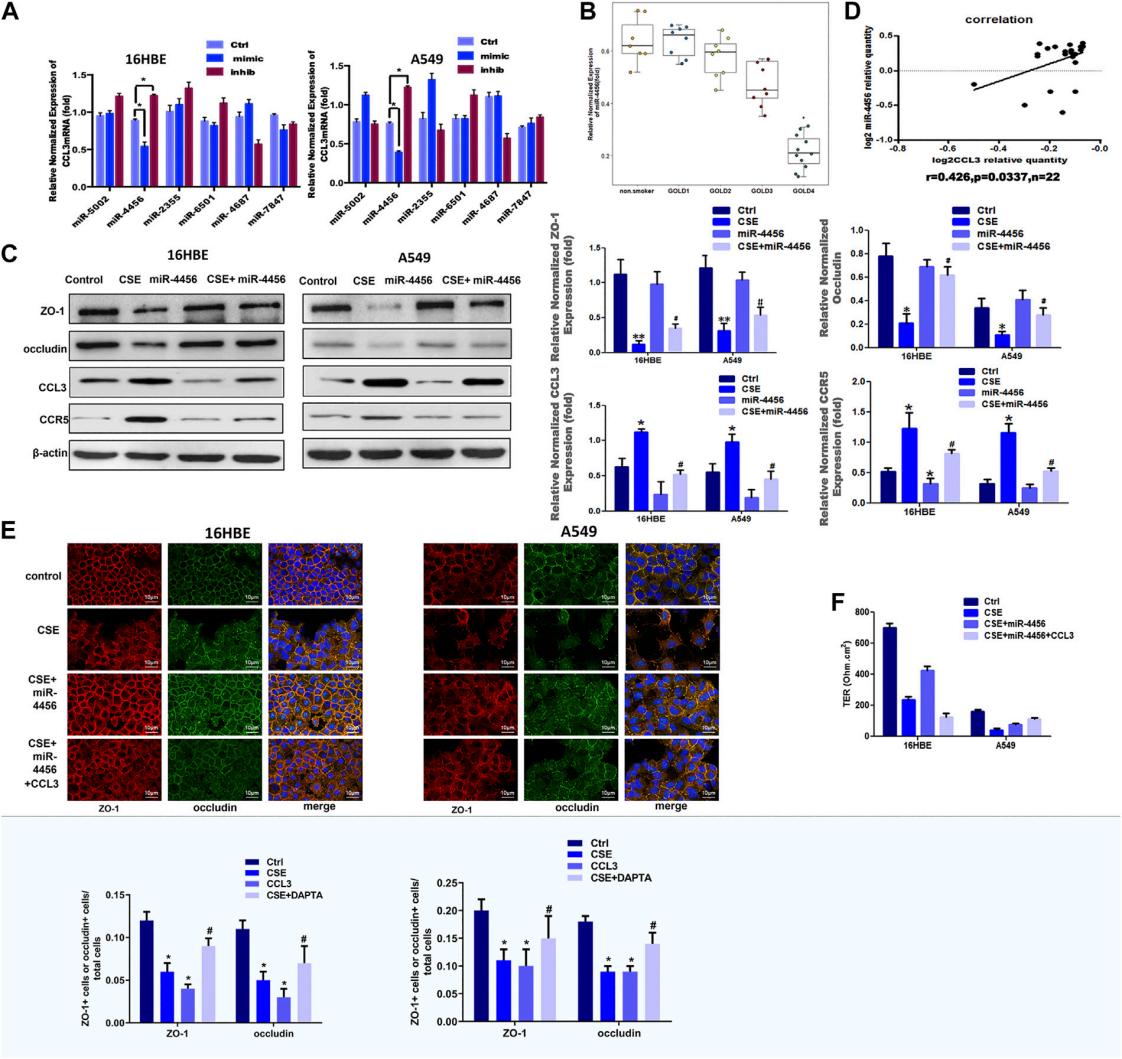
Identification of miR-4456 as an upstream signal of CCL3 induced-tight junction injury **(A)** Real-time PCR screen in 16-HBE cells (left panel) and A549 cells (right panel) for miRNAs that could target CCL3 mRNA. CCL3 mRNA expression levels were normalized to GAPDH expression. *n* = 3, *p < 0.05, vs. the control group. The expression levels were normalized to the expression of GAPDH **(B)** Baseline expression of miR-4456 according to COPD grade. *p < 0.05 vs. the non-smoker group ((*n* = 48. non-smokers:8, GOLD 1:9, GOLD2: 9, GOLD3:10, GOLD4:12) **(C)** Western blot analysis of ZO-1, occludin, CCL3, and CCR5 protein expression in the four groups: 1) Control (PBS 10 mg ml^−1^), 2) CSE (10 mg ml^−1^), 3) miR-4456, and 4) CSE (10 mg ml^−1^)+miR-4456 mimics in 16HBE cells and A549 cells. n = 3, **p < 0.01, vs. the control group, *p < 0.05, vs. the control group, #*p* < 0.05, vs. the CSE group. Protein expression was normalized to β-actin **(D)** Correlation analysis of miR-4456 and CCL3 mRNA levels by Pearson correlation coefficient from a total of 22 COPD peripheral blood samples (*n* = 22) **(E)** Tight junction proteins ZO-1 (red), occludin (green) and merged (blue + orange) were stained by immunofluorescence in the four groups: 1) Control (PBS 10 mg ml^−1^), 2) CSE (10 mg ml^−1^), 3) CSE (10 mg ml^−1^)+miR4456, and 4) CSE (10 mg ml^−1^)+miR4456 mimics + CCL3 (10 mg ml^−1^) in 16HBE cells (left panel) and A549 cells (right panel). Presented as means ± standard error. *n* = 3, *p < 0.05, vs. the control group, **p < 0.01, vs. the control group, #*p* < 0.05, vs. the CSE group,△*p* < 0.05, vs. the CSE + miR-4456 group. Data are representative of three independent experiments. Scale bar = 10 µm **(F)** TER in the four groups same as Figure-3Eis shown as a function test on 7 days after plating the airway epithelial cells on coated permeable filters. Presented as means ± standard error. *n* = 3, *p < 0.05, vs. the control group, **p < 0.01, vs. the control group, #*p* < 0.05, vs. the CSE group,△*p* < 0.05, vs. the CSE + miR-4456 mimics group.

Recent evidences have highlighted an emerging role for miRNAs as the crucial regulators of epithelial barrier functions (Neudecker et al., 2017; Zhu et al., 2018). We then examined whether miRNAs were involved in the CCL3 dependent TJs injury. We first identified six candidate miRNAs (miR-5002, miR-4456, miR-2355, miR-6501, miR-4687 and miR-7847) that might suppress CCL3 expression through its 3′UTR target by searching multiple databases (TargetScan, miRDB, and microRNA.org). We examined the overexpression effects of these miRNAs on CCL3 expression in 16HBE and A549 cell lines. We found miR-4456 overexpression led to a significant decrease of CCL3 in both cell lines (Figure 3A). Furthermore, the miR-4456 inhibitor (5 nM, MIN0000090, Qiagen) increased CCL3 mRNA in both cell lines (Figure 3A). In very severe COPD, the expression of mir-4456 was lower than that of the normal control group, but there was no significant difference between patients with mild and moderate COPD (Figure 3B). Cells pretreated with miR-4456 significantly correlated with the effect of CSE on the expression of ZO-1, occludin, CCL3 and CCR5 protein (Figure 3C). Besides, we examined miR-4456 and CCL3 expression in blood samples of GOLD3-4 stage COPD and found a significant positive correlation (*r* = 0.426, *p* = 0.0337) between miR-4456 expression and CCL3 expression in 22 specimens (Figure 3D). So we further investigated the correlation of miR-4456 and CCL3 expression in very severe COPD tissues (*n* = 76, Supplementary Figure S1 and Supplementary Table S1), which showed a significant negative correlation (*r* = −0.8813, *p* < 0.0001), indicating a potential suppressive role of miR-4456 in the progression of severe COPD. We then applied an immunofluorescence assay to examine the effect of miR-4456 in TJs, and found that miR-4456 significantly suppressed the destruction of TJs induced by CSE. Morever, overexpression of miR-4456 with CCL3 could not suppress the destruction, indicating that miR-4456 improved the CSE induced TJs injury through a CCL3 dependent way in 16HBE cells and A549 cells (Figures 3E,F). Together, these results demonstrated that miR-4456 could improve TJ injury by downregulating CCL3 expression.

### MiR-4456 Suppresses CCL3 Expression via the 3′UTR

**FIGURE 4:**
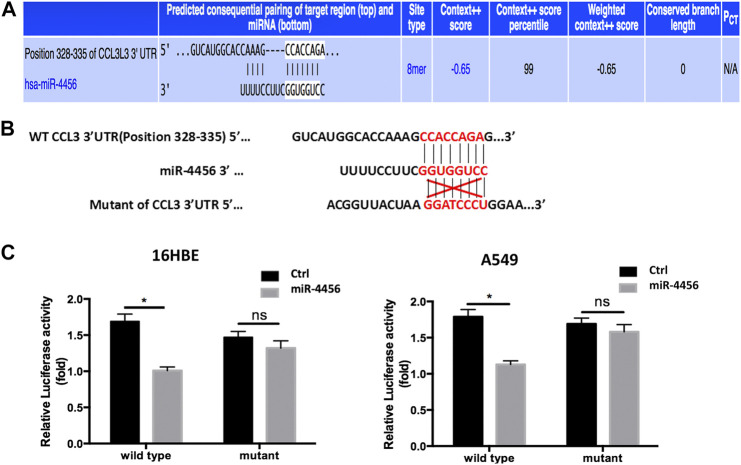
miR-4456 suppresses CCL3 expression through 3ʹUTR of CCL3 **(A,B)** The predicted duplex formation between wild human type (WT) CCL3 3ʹ-UTR and human miR-4456 **(C)** luciferase assays were performed to detect the regulation of miR-4456 on WT and mutant CCL3-3ʹUTR. Assays were performed on CCL3-wt.3ʹUTR ± miR-4456, or CCL3-mut.3ʹUTR ± miR-4456 in 16-HBE cells (left panel) and A549 cells (right panel). Data are represented as the mean ± standard deviation. *n* = 3, *p < 0.05, compared to controls.

To further dissect the molecular mechanisms through which miR-4456 decreased CCL3 expression, we identified one predicted miRNA-responsive-element that matched the seed sequence of miR-4456 in the 3′UTR of the CCL3 gene (Figure 4A). We inserted a 359 bp fragment from the CCL3 3′UTR with the predicted miR-4456 target site into a dual-luciferase reporter backbone (psiCHECK™-2) downstream of the Renilla luciferase open reading frame (ORF). Simultaneously, we also included a mutated version at the predicted target site (Figure 4B). As expected, the luciferase assay revealed that depletion of miR-4456 significantly increased luciferase activity in 16HBE cells, while the addition of miR-4456 markedly decreased luciferase activity in A549 cells transfected with wild type TR4 3′UTR. However, these effects could not be observed when the mutant CCL3 3′UTR was transfected into these cells (Figure 4C), suggesting that miR-4456 could directly and specifically regulate CCL3 expression through binding to the CCL3 3′UTR.

### *In vivo* Mice Studies Confirmed the Role of CCL3 and miR-4456 in COPD

**FIGURE 5:**
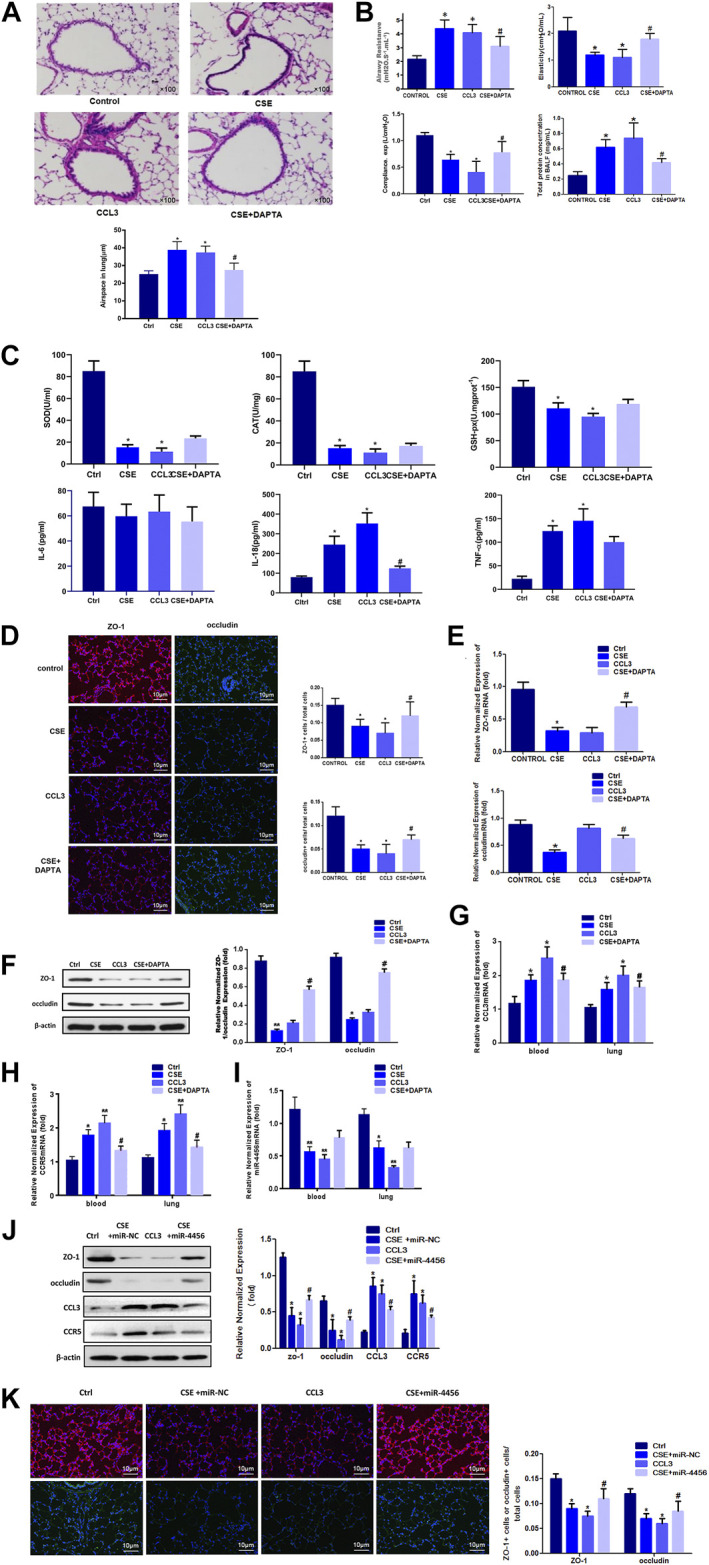
*In vivo* mouse studies confirm the role of CCL3 and miR-4456 in COPD **(A)** H&E staining confirms the macroscopic appearance of pulmonary tissue in the four groups: 1) Control (Ctrl), 2) CSE, 3) CCL3, and 4) CSE + DAPTA. Quantification of the alveolar space is in the lower panels. *n* = 8,*p < 0.05, vs. the control group, #*p* < 0.05, vs. the CSE group **(B)** Airway resistance, Elasticity, Compliance, and total BALF proteins were detected in the four groups same as Figure 5A *n* = 8,*p < 0.05, vs. the control group, #*p* < 0.05, vs. the CSE group **(C)** Inflammatory and oxidative stress markers in the four groups same as Figure 5A *n* = 8,*p < 0.05, vs. the control group, #*p* < 0.05, vs. the CSE group **(D)** Tight junction proteins ZO-1 merge (red and blue), and occludin merge (green and blue) were stained using immunofluorescence in the four groups same as Figure 5A. Data are representative of three independent experiments. Scale bar = 10 µm *n* = 8, *p < 0.05, vs. the control group, #*p* < 0.05, vs. the CSE group **(E)** Real-time PCR assays were performed in the blood and lung to detect ZO-1 and occludin mRNA before and after CSE treatment in the four groups same as Figure 5A. Data are represented as the mean ± standard deviation. *n* = 8, *p < 0.05, vs. the control group. #*p* < 0.05, vs. the CSE group. The expression levels were normalized to the expression of GAPDH, *n* = 3 **(F)** Western blot for ZO-1 and occludin protein expression in the four groups same as Figure 5A *n* = 8, *p < 0.05, vs. the control group, **p < 0.01, vs. the control group, #*p* < 0.05, vs. the CSE group. Protein expression was normalized to β-actin **(G)** Real-time PCR assays were performed in the whole blood and lung to detect CCL3 mRNA expression in four groups same as Figure 5A. Data are represented as the mean ± standard deviation. *n* = 8, *p < 0.05, vs. the control group, #*p* < 0.05, vs. the CSE group. The expression levels were normalized to the expression of GAPDH **(H)** Real-time PCR assays were performed in the whole blood and lung to detect CCR5 mRNA expression in four groups same as Figure 5A. Data are represented as the mean ± standard deviation. *n* = 8, *p < 0.05, vs. the control group, **p < 0.01, vs. the control group, #*p* < 0.05, vs. the CSE group. The expression levels were normalized to the expression of GAPDH **(I)** Real-time PCR assays were performed in the whole blood and lung to detect miR-4456 expression in four groups same as Figure 5A. Data are represented as the mean ± standard deviation. *n* = 8, *p < 0.05, vs. the control group, **p < 0.01, vs. the control group. The expression levels were normalized to the expression of 5S RNA and/or U6 **(J)** Western blot for ZO-1 and occludin protein expression in the four groups: 1) Control (Ctrl), 2) CSE, 3) CCL3, and 4) CSE + miR-4456. *n* = 8, *p < 0.05, vs. the control group, #*p* < 0.05, vs. the CSE group. Protein expression was normalized to β-actin **(K)** Tight junction proteins ZO-1 merge (red and blue), and occludin merge (green and blue) were stained using immunofluorescence in the four groups same as Figure 5J. Data are representative of three independent experiments. Scale bar = 10 µm *n* = 8, *p < 0.05, vs. the control group, #*p* < 0.05, vs. the CSE group.

Because cigarette smoke (CS) was critical to the pathogenesis of COPD, we then accessed the expression of CCL3 in a mouse model of CS-induced COPD. The mouse developed an emphysematous phenotype after 24 weeks of CSE, showing enlargement of the air spaces accompanied by the destruction of the alveolar architecture (Figure 5A, upper). To quantify the presence and severity of emphysema, we determined the enlargement of alveolar spaces by measuring the mean linear intercept (Lm). Compared with air-control mice (25.2 ± 1.8 μm), significant alveolar space enlargement was observed in mice exposed to CS (38.9 ± 4.6 μm); CCL3 had a similar effect to that of CSE (37.4 ± 3.6 μm), and DAPTA could reverse the effect of CCL3 (27.5 ± 3.9 μm) (Figure 5A, lower). We next detected the mouse lung function: the airway resistance of the CSE or CCL3 groups was significantly increased compared to the control group, while elasticity and compliance was increased, and total protein concentrations were elevated. Those changes induced by CSE could be reversed by DAPTA. (Figure 5B). Figure 5C showed that CSE and CCL3 increased the IL-18 and TNF-α levels compared with the controls and decreased SOD, CAT, and GSH-Px levels (all *p* < 0.05). Only IL-18 was decreased by DAPTA compared with the CSE group (*p* < 0.05), IL-6 was not influenced by CSE or CCL3. Furthermore, there was significant epithelial TJ injury in the CSE and CCL3 groups compared with the control group, while DAPTA could reverse the effect of CSE in mice (Figure 5D). Accordingly, CSE or CCL3 decreased the mRNA expression of ZO-1 and occludin, while DAPTA partly abolished this effect (Figure 5E). Similar effects were observed at the protein level by western blotting (Figure 5F). Consistent with the results in COPD patients, we found that CCL3 and CCR5 mRNAs were significantly upregulated in the lung of mice after CSE treatment when compared with air-control mice, while DAPTA could reverse the effect of CSE in mice, and similar results were also found in the blood of the mice (Figures 5G,H). Similarly, there was a significant decrease in miR-4456 mRNA expression in blood and lung tissues of mice exposed to CS, but DAPTA could not reverse the effect of CSE to miR-4456 (Figure 5I). CSE or CCL3 decreased the protein expression of ZO-1 and occludin, while miR-4456 partly blocked the effect of CSE (Figures 5J,K).

Taken together, our results from the *in vivo* mouse model were consistent with that of the *in vitro* cell line studies, demonstrating that CCL3 promoted epithelial TJ injury through the miR-4456-CCL3-CCR5 pathway.

## Discussion

The lung tissues of patients with COPD are affected by the local immune and inflammatory environment, but the systemic immune and inflammatory environment also play a role in the development of COPD. Studies have proved that PBMCs had a crucial role in COPD (Bahr et al., 2013, Tan, Xuan et al., 2016). We demonstrated that CCL3 was a significantly increased inflammatory cytokine in the PBMCs from patients with severe COPD. CCL3 downregulated the expression of ZO-1 and occludin, thus inducing severe injury of TJs both *in vivo* and *in vitro*. CSE could upregulate the mRNA and protein level of CCL3, while CCR5 antagonist DAPTA could reverse this effect of CSE. Furthermore, miR-4456 could suppress the effects of CCL3 on TJs by binding to the CCL3 3′-UTR. Our results demonstrated that CSE induced injury to airway epithelium TJs via the miR-4456/CCL3/CCR5 pathway.

The loss of lung function in patients with COPD and emphysema is associated with a high percentage of CD4^+^ and CD8^+^ T lymphocytes that express receptors CCR5 and CXCR3, but not CCR3 or CCR4 (both markers of T helper one cells) (Grumelli et al., 2004). Previous studies showed that CCR1 and CCR5 acted together with CCL3 to play a role in COPD. Since using CCR1 antagonists could not treat COPD (Kerstjens et al., 2010), we thus applied CCR5 antagonist in our research. Previous studies have shown that CCR3/CCR5 expression was correlated with COPD severity (Freeman et al., 2007). Chronic CSE significantly increased CCR5 expression, and the number and extent of peribronchial lymphoid follicles (Bracke et al., 2007). It could also induce airspace enlargement in wild-type mice. Conversely, inflammatory cells in BALF and peribronchial lymphoid follicles were all significantly attenuated, and airspace enlargement was reduced in CCR5 knockout (KO) mice (Bracke et al., 2007). Still, CCR5 deficiency did not affect CSE-induced airway wall remodeling (Bracke et al., 2007). The follow-up studies showed that CCL3/CCR5 contributed to increased numbers of macrophages and T-cells in the lungs of patients with COPD (Ravi et al., 2014; Costa et al., 2016). Recently, it has been suggested that IL-8 overexpression increased the expression of CCL3 and reduced the expression of Claudin 18 and F11r, inducing damage to the epithelial organization and leading to leaky TJs (Reynolds et al., 2018). These results showed that CCL3/CCR5 specifically caused lung damage through persistent inflammation and damaged TJs, but not lung remodeling. Our results demonstrated that CCL3 induced significant injury to TJs through its receptor CCR5, which was in accordance with previous CCL3/CCR5 studies in COPD. Furthermore, CSE could upregulate the expression of CCL3 mRNA and protein, and CCR5 antagonist DAPTA could reverse the effect of CSE both *in vivo* and *in vitro*.

It was well established that miRNAs were relevant to the pathogenesis of COPD (Ezzie et al., 2012). A previous study has shown that in human bronchial airway epithelium, miRNA expression was affected by smoking, since most miRNAs were found to be downregulated in current-smokers (Schembri et al., 2009). Exosomal miRNAs released from macrophages could lead to a series of events in recipient alveolar epithelial cells, resulting in impairment of tight junction barrier integrity and mitochondrial bioenergetics (Zhang et al., 2020). These changes in the alveolar microenvironment increased the susceptibility to lung infection and injury (Yuan et al., 2019). Nevertheless, those exosomal miRNAs were not assessed in the present study. Growing evidence indicated that lung epithelial damage resulted in impairment of the tight junction barrier, which disrupted homeostasis of the tissue microenvironment. The junctional adaptor protein ZO-1 was reported to have a central regulatory role in epithelial barrier formation (Nazli et al., 2010; Fernandes et al., 2018). Taking advantage of the data from multiple databases (TargetScan, miRDB, and microRNA.org), we screened miRNAs and found that miR-4456 could suppress the effect of CCL3/CCR5 on TJs through binding to the 3′-UTR of CCL3. In addition, there was a significant decrease in miR-4456 mRNA expression both in lung tissues from CS-exposed mice. In this study, we showed that the crosstalk between PBMCs and lung epithelial cells impaired epithelial barrier integrity through miR-4456/CCL3/CCR5/ZO-1 and occludin. The present study suggested that targeting miR-4456 might be of therapeutic value to enhance lung epithelial barrier in COPD, and miR-4456 mRNA might be an indicator of the severity of inflammation in COPD. Future investigation should be done to further understand the roel of miR-4456 in the pathogenesis and immune regulation of COPD.

There were very few effective disease-modifying treatments for COPD, and most treatments were merely symptomatic treatments (Barnes 2018). Identification of new mechanisms that could suppress the inflammatory response in COPD was urgently needed for the development of better therapies. Importantly, since we found that CCL3 can promote TJ injury via CCR5, and miR-4456 can suppress CCL3 both *in vivo* and *in vitro*, thus targeting these genes might lead to novel therapies for COPD. Nevertheless, there were probably hundreds of miRNAs that are upregulated or downregulated in COPD (Ezzie et al., 2012; Osei et al., 2015; Sato et al., 2015; Szymczak et al., 2016; Conickx et al., 2017; Keller et al., 2018), and the aim of the present study was only to examine those that could modulate the CCL3/CCR5 axis. In addition, although A549 cells were used to study alveolar epithelial cells (Akram et al., 2013; Mortaz et al., 2017; Somborac-Bacura et al., 2018), they were malignant cells that might not reflect reality. Future studies should be done to examine a wide panel of miRNAs, and also to deline at the effects of circulating miRNAs vs. those of miRNAs produced locally in the lungs. Furthermore, larger sample size and patients with different stages required to be explored, since in our study, the correlation of miR-4456 and CCL3 expression in Stage 3–4 COPD was contradictory with two different sample size, which might resulte from small sample size or flexible expression of miRNAs in the peripheral blood of different stages.

## Conclusion

MiR-4456 played an important role in the epithelial TJs impairment of COPD. miR-4456/CCL3/CCR5 was a potential therapeutic pathway for the treatment of COPD.

## Data Availability

The datasets presented in this study can be found in online repositories. The names of the repository/repositories and accession numbers can be found in the article/Supplementary Material.

## Abbreviations

CCL3, chemokine (C-C motif) ligand 3; CCR5, C-C chemokine receptor type 5; COPD, chronic obstructive pulmonary disease; CSE, cigarette smoke exposure; GOLD, Global Initiative for Chronic Obstructive Lung Disease; MIP-1α, macrophage inflammatory protein-1α; PBMC, Peripheral blood mononuclear cells; ZO-1, zona occludens-1
