# Supplementary material for: miR-4456/CCL3/CCR5 Pathway in the Pathogenesis of Tight Junction Impairment in Chronic Obstructive Pulmonary Disease
Source: Front Pharmacol. 2021 Apr 19;12:551839. doi: 10.3389/fphar.2021.551839 (PMC8089484; doi:10.3389/fphar.2021.551839)
Supplement: Supplementary file 1 [file table1.pdf]

Supplementary table1： Clinical characteristics of the subjects involved in the studies

Characteritics	GOLD3	GOLD4
**Number of subjects**	9	67
**Age (years)**	77.6±15.3	75.3±18.8
**Sex, male (female)**	9(3)	60(7)
**Pack-years**	42.5±5.3	46.7±6.8
**FEV1 (% predicted)**	48.4±6.9	25.6±2.8
**FEV1/FVC (%)**	42.1±5.3	21.4±4.1
**Under ICU**	6	61

Notes: Values are expressed as mean±SD.

Abbreviations: FEV1 (% predicted), forced expiratory volume in 1 second as percentage of percentage of predicated value; FVC, forced vital capacity.
