# Supplementary figures and images for: miR-4456/CCL3/CCR5 Pathway in the Pathogenesis of Tight Junction Impairment in Chronic Obstructive Pulmonary Disease
Source: Front Pharmacol. 2021 Apr 19;12:551839. doi: 10.3389/fphar.2021.551839 (PMC8089484; doi:10.3389/fphar.2021.551839)

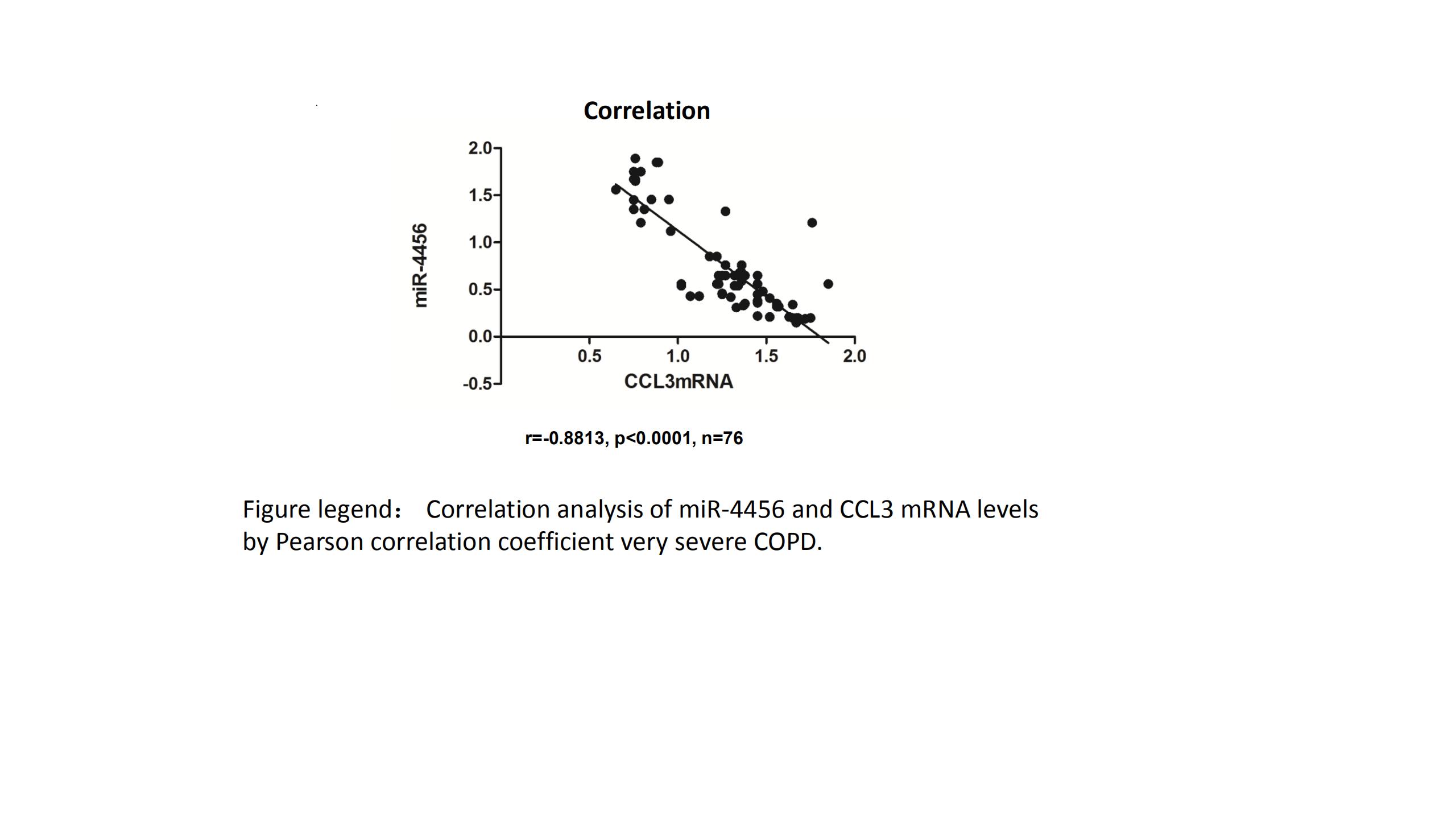

Supplement: Supplementary file 2 [file image1.tif]
